# The neovascularization effect of dedifferentiated fat cells

**DOI:** 10.1038/s41598-020-66135-1

**Published:** 2020-06-08

**Authors:** Hirofumi Watanabe, Shumpei Goto, Reona Kato, Shogo Komiyama, Yuki Nagaoka, Tomohiko Kazama, Chii Yamamoto, Yuxin Li, Noriyoshi Konuma, Kazuhiro Hagikura, Taro Matsumoto

**Affiliations:** 1 Department of Pediatrics and Child Health, Nihon University School of Medicine, Tokyo, Japan; 2 Department of Pediatric Surgery, Nihon University School of Medicine, Tokyo, Japan; 3 Department of Functional Morphology, Division of Cell Regeneration and Transplantation, Nihon University School of Medicine, Tokyo, Japan

**Keywords:** Mesenchymal stem cells, Regeneration

## Abstract

Mature adipocyte-derived dedifferentiated fat (DFAT) cells can be prepared efficiently and with minimal invasiveness to the donor. They can be utilized as a source of transplanted cells during therapy. Although the transplantation of DFAT cells into an ischemic tissue enhances angiogenesis and increases vascular flow, there is little information regarding the mechanism of the therapeutic angiogenesis. To further study this, mice ischemic hindlimb model was used. It was confirmed that in comparison with the adipose derived stem cells and fibroblasts, the transplantation of DFAT cells led to a significant improvement in the blood flow and increased mature blood vessel density. The ability of DFAT cells to secrete angiogenic factors in hypoxic conditions and upon co-culture with vascular endothelial cells was then examined. Furthermore, we examined the possibility that DFAT cells differentiating into pericytes. The therapeutic angiogenic effects of DFAT cells were observed by the secretion of angiogenic factors and pericyte differentiation by transforming growth factor β1 signalling via Smad2/3. DFAT cells can be prepared with minimal invasiveness and high efficiency and are expected to become a source of transplanted cells in the future of angiogenic cell therapy.

## Introduction

Peripheral artery disease (PAD) is a condition based on stenosis or occlusion lesions of the lower limb arteries, most of which are caused by atherosclerosis obliterans (ASO). A second cause of PAD is Buerger’s disease, caused by thromboangiitis. While the number of patients with Buerger’s disease has declined, the number of patients with ASO continues to rise with over 200 million cases reported worldwide^1^. A severe form of PAD is critical limb ischemia (CLI), the incidence of which ranges from 500 to 1,000 cases per million annually in European and North American populations. It is estimated that approximately 250,000 patients undergo major amputation of the lower extremities annually^2^. The prognosis of patients with CLI is extremely poor, leading to 30% large leg amputation and 25% death in patients annually^2^. However, 25 to 50% of CLI patients do not have any indication for revascularization therapy, due to the lack of autologous vein grafts, extensive peripheral arterial lesions, or serious complications^2^. An effective method for the treatment of CLI is yet to be determined despite the high medical necessity.

More recently, various cell-based therapies such as bone-marrow mononuclear cells (BM-MNCs), bone-marrow mesenchymal stem cells (BM-MSCs), adipose-derived stem cells (ASCs), and endothelial progenitor cells (EPCs), have been studied as potential sources of the therapeutic angiogenesis of CLI^3–5^. Of these, mesenchymal stem cells (MSCs) and ASCs were less tumorigenic compared to embryonic stem cells (ESCs) and induced pluripotent stem cells (iPSCs) and could be safely transplanted due to its genetic stability^6^. Indeed, there are almost 1,000 registered clinical trial with MSCs have been conducted worldwide^7^. MSCs and ASCs secrete various angiogenic factors such as fibroblast growth factor 2 (FGF2), vascular endothelial growth factor (VEGF), angiogenin, hepatocyte growth factor (HGF) and platelet-derived growth factor BB (PDGF-BB), and have the ability to differentiate into vascular endothelial cells and pericytes^8^. Clinical trials of cell therapy using bone marrow MSC^9^ and ASC^10^ have also been conducted on patients with CLI, and their safety and efficacy have been reported. However, according to a recent meta-analysis of 27 randomized controlled trials^11^, no significant improvement in the ability to salvage major limbs was reported. Additionally, the long-term effectiveness had not been clarified. Hence, a cell transplant therapy is yet to be established as the standard treatment protocol for CLI. Matsumoto *et al*. reported that dedifferentiated fat (DFAT) cells, which are made from cultures of mature adipocytes, could have multipotency like MSCs with higher purity and preparation efficiency than other somatic stem cells^12^. In addition, it was confirmed that DFAT cells exhibited a neovascularization effect^13,14^. DFAT cells are now being sought out as a newer therapeutic cell source. But its effects, and the mechanism by which it acts, are still unknown. In this study, the effects of transplantation of DFAT cells in the mouse ischemic hindlimb model was evaluated. Moreover, the interactions of DFAT cells with endothelial cells, and the differentiation potential of DFAT cells to the pericytes, was assessed *in vitro*.

## Materials and methods

### Animals

C57BL/6 mice, severe combined immunodeficiency disease (SCID) mice, C57BL/6-Tg (CAG-EGFP) mice and Fabp4-Cre/LacZ-ROSA (R26R) mice were obtained from the Jackson Laboratory (ME, USA). Animal experiments were conducted in accordance with the policies of Nihon University School of Medicine Animal Experiments Committee (Approval number: AP10M023, AP10M025, AP16M020).

The rearing and use of genetically modified mice for experimental purposes was conducted with the approval of the Nihon University Genetic Recombination Experiment Safety Committee (Approval number: 2009 Med3, 2010Med11, 2011Med3, 2011Med10).

### Isolation and cell culture

DFAT cells and ASCs were obtained in accordance with a previously established method^12^. Briefly, the adipose tissue sample obtained from mice was treated with collagenase and centrifuged, and ASCs were prepared from the precipitated stromal vascular fraction. DFAT cells were prepared from lipid-filled mature adipocytes and cultured in ceiling cultures. The growth medium was CSTI-303MSC (Cell Science & Technology Institute, Miyagi, Japan) containing 20% Foetal Bovine Serum (FBS). Media replacement was performed every 3–4 days. Cells within the fourth passage were used for experiments.

mCherry-labelled Mile Seven 1 (MS1) cells were obtained from the Karolinska Institute (Sweden). Mouse primary dermal fibroblasts were obtained from Cell Biologics (Chicago, U.S.A). The growth medium used was Dulbecco’s Modified Eagle Medium (DMEM) (Invitrogen, CA, USA) containing 10% FBS. Media replacement was performed every 3–4 days.

### Cell differentiation

For osteogenic differentiation, DFAT cells were cultured for 3 weeks in DMEM containing 10% FBS, 100 nM dexamethasone, 10 mM β-glycerophosphate (Sigma-Aldrich, MO, USA), and 50 μM L-ascorbic acid-2-phosphate (Sigma-Aldrich). Differentiated cells were stained with Alizarin red S (Sigma-Aldrich) after being fixed in 4% paraformaldehyde. For chondrogenic differentiation, DFAT cells were seeded at a density of 2 × 10^5^ cells per pellet, in 15 cm^3^ conical tubes. Cells were gently centrifuged at 150 g for 5 min. DFAT cells were cultured for 3 weeks in DMEM containing 1% FBS, 50 μM L-ascorbic acid-2-phosphate, 40 μg/ml proline (Sigma-Aldrich), 100 μg/ml pyruvate (Sigma-Aldrich), 10 ng/ml transforming growth factor (TGF)-β3 (R&D Systems, MN, USA), and 1x insulin-transferrin-selenium-X (ITS) (Invitrogen). The pellet was fixed in 4% paraformaldehyde and then observed under a stereomicroscope. For adipogenic differentiation, DFAT cells were incubated for 3 weeks in 10% FBS DMEM, 1 μM dexamethasone (Sigma-Aldrich), 0.5 mM isobutylmethylxanthine (Sigma-Aldrich), 1x ITS. Differentiated cells were stained with Oil red O (Sigma-Aldrich) after being fixed in 4% paraformaldehyde.

### Flow cytometry

Fluorescence-activated cell sorting (FACS) or flow cytometric analysis was performed to characterize the DFAT cells. The following antibodies were used in the mesenchymal stem cell marker antibody panel (R&D Systems, MN, USA): anti-Sca1-APC, anti-CD11b-APC, anti-CD29-APC, anti-CD45-APC, anti-CD73-APC, anti-CD105-APC, and anti-CD106-APC. Cells were analysed with a FACSAria flow cytometer using the CellQuest Pro software, version 5.1(Becton Dickinson, NJ, USA). The number of positive cells was compared with the signal obtained for the corresponding immunoglobulin isotypes.

### Enzyme-linked immunosorbent assay (ELISA)

DFAT cells, ASCs and fibroblasts were cultured under normal oxygen and hypoxic conditions (1% O_2_) for 48 hours, and the concentrations of VEGF-A and HGF in the culture supernatant were measured. The concentrations of culture supernatant were analysed using a sandwich ELISA method according the manufacturer’s protocol (MMV00, MHG00) (R&D Systems).

### Immunofluorescence analysis

NG2 antibody (1:200) (Merck Millipore, MA, USA),α smooth muscle actin (ASMA) antibody (1:200) (Dako, CA, USA), and isolectin B4 (IB4) (1:100) (Vector Laboratories, CA, USA) were used for the primary antibody reaction at 4 °C overnight. For the secondary antibody reaction, Alexa-647-labelled donkey anti-rabbit IgG antibody (1:400) (Thermo Fisher Scientific, MA, USA) and Alexa-594-labelled donkey anti-mouse IgG antibody (1:400) (Thermo Fisher Scientific) were used. Nuclear staining was performed using 5 μg/ml Hoechst 33342. The samples were visualized using a confocal laser scanning microscope (Fluoview FV10i, Olympus, Tokyo, Japan) and a fluorescence microscope (BZ-X710, Keyence, Osaka, Japan).

### RNA isolation and real-time reverse transcription-polymerase chain reaction (RT-PCR)

The total RNA was extracted from cells using an RNeasy Micro kit (Qiagen, Hilden, German) according to the manufacturer’s protocol. The reverse transcription reactions were conducted using high capacity cDNA Reverse Transcription kits (Applied Biosystems, CA, USA), yielding cDNA. TGF-β1 (Mm_01178820), Platelet-derived growth factor (PDGF)-BB (Mm_00440677), HGF (Mm_00440677), VEGF-A (Mm_00440677), fibroblast growth factor (FGF) 2 (Mm_00440677), Angiopoietin-1 (Ang1) (Mm_00440677), and the pericyte markers NG2 (Mm_00507257), ASMA (Mm_00725412), RGS5 (Mm_00654112), and PDGFRβ (Mm_00435546) were used as Taqman primers. TaqMan Fast Advanced Master Mix Product Inserts (Applied Biosystems) and a StepOnePlus real time PCR system (Applied Biosystems) were used for the PCR reactions. Glyceraldehyde-3-phosphate dehydrogenase (GAPDH) expression was measured along with a reference standard. Each sample was analysed by relative quantification (comparative C_T_ method) with regard to GAPDH.

### *In vitro* experiments

For the co-culture assay, green fluorescent protein (GFP)-labelled DFAT cells were cultured alone (control group), and co-cultured with MS1 cells directly (direct co-culture group), or indirectly (indirect co-culture group) using a cell culture insert with 0.4 μm pores (Corning, NY, USA). DMEM with 5% FBS was used as the culture medium. After culturing for 72 hours, the cells were collected and the total RNA was extracted for RT-PCR. In RNA analysis, in order to collect DFAT cells from MS1 cells separately, each type of cells was plated on both faces of a cell culture insert with 0.4 μm pores in direct co-culture group. After 96 hours of co-culturing, the cells were fixed and immunofluorescence staining was performed.

For the TGF-β1 assay, GFP-labelled DFAT cells were cultured in 5% FBS DMEM containing 50 ng/ml human recombinant TGF-β1 (PeproTech, NJ, USA). The Smad2/3 inhibition experiments were performed by adding 5 μM PD169316 (Sigma-Aldrich) or dimethyl sulfoxide (DMSO) (Sigma-Aldrich) into the culture medium with TGF-β1. The total RNA was extracted for RT-PCR analysis after 72 hours, and immunofluorescence staining samples were fixed after 96 hours. In addition, GFP-labelled DFAT cells were cultured with MS1 cells in 5% FBS DMEM for the TGFβ1 inhibition experiments. PD169316 (5 μM), TGFβ1 neutralizing antibody (25 μg/ml 1D11.16.8) (GeneTex, CA, USA), or DMSO were added to the culture medium. After 96 hours of co-culturing, each treatment group was fixed and immunofluorescence staining was performed.

For the tube formation assay using MS1 cells, DsRed-labelled MS1 cells (MS1 group) and DsRed-labelled MS1 cells with GFP-labelled DFAT cells (MS1 + DFAT group) were attached to collagen beads (Cytodex3, GE Healthcare). The collagen beads were then embedded into the collagen gel (collagen type I rat tail, Corning, NY, USA). The MS1 and MS1 + DFAT groups were cultured in 10% FBS DMEM. On the 7th day of culturing, the cells were fixed and nuclear staining with 5 μg/ml Hoechst 33342 (Invitrogen) was performed. The tube formations were observed using the confocal laser scanning microscope (Fluoview FV10i) and the fluorescence microscope (BZ-X710). The tube length and area were quantified using Image J software, version 1.52a (imaagej.nih.gov)^15^. Another tube formation assay using human umbilical vein endothelial cells (HUVECs) was performed using an angiogenesis kit (Kurabo, Osaka, Japan) according to the manufacturer’s instructions. Briefly, DFAT cell conditioned medium was collected after culturing the cells under normal oxygen or hypoxic conditions (1% O_2_) for 48 hours. HUVECs were co-cultured with human fibroblasts as feeder cells in 24-well plates with or without DFAT cell conditioned medium diluted 1:1 with the assay medium (Kurabo). The medium was replaced every 3 days. After 11 days of culture, cells were fixed and immnuostained with mouse monoclonal anti-human CD31 antibody (1:4000, Kurabo) followed by FITC-labelled goat anti-mouse IgG to visualize tube-like structures of HUVECs. The total tube length and total tube area in three field/well were quantified using Angiogenesis Image Analyzer software, version 2.0.4 (Kurabo). Each sample was tested in triplicate wells.

### Matrigel plug assay

GFP-labelled DFAT cells (1 × 10^6^) were mixed with 250 μl DMEM with 5% FBS and 250 μl ice-cold Matrigel (Corning Matrigel 354248, Corning, NY, USA). It was then subcutaneously injected into the cervical area of 10-week-old male C57BL/6 mice with an ice-cold syringe and a 23 G needle. The Matrigel was extracted 21 days after transplantation, fixed with 4% paraformaldehyde, embedded in paraffin and sectioned onto slides. The slides were stained using ASMA (1:100), GFP (1:100), von Willebrand factor (vWF) (1:100), and 5 μg/ml Hoechst (1:500) as primary antibodies, and Alexa Fluor 488 (1:200) and Alexa Fluor 594 (1:200) as secondary antibodies. The tissue samples were observed using the confocal laser scanning microscope (Fluoview FV10i) and the fluorescence microscope (BZ-X710).

### Western blot analysis

Mouse DFAT-D1 cells in a 60-mm dish were pretreated with PD169316 for 1 hour, followed by incubation with 50 ng/ml of TGFβ1 for 1 hour. Whole cell protein extracts were prepared by lysing cells in cell lysis buffer containing 50 mM Tris, 150 mM NaCl, 1% Triton X-100, 0.1% SDS, 0.1% sodium deoxycholate, protease/phosphatase inhibitor cocktail (5872S, Cell signaling Technology, MA, USA). Western blot analysis was performed by loading 50 µg of cell lysate using e-PAGEL polyacrylamide gel (E-T10L, ATTO, Tokyo, Japan), electrophoresis system (AE-6530, ATTO), Rainbow molecular marker (RPN800E, GE Healthcare Life Sciences, England) and semi-dry blotting system (HorizeBLOT, ATTO). Proteins were transferred to a PVDF membrane (WSE-4051, ATTO) and blocked with 3% bovine serum albumin (BSA) for 1 hour at room temperature. The blots were incubated with rabbit anti-TGFβ receptor 1 (TGFBR1) antibody (PA5-32631, Thermo Fisher, IL, USA), rabbit anti-phospho-TGFBR1 antibody (PA5-40298, Thermo Fisher), rabbit anti-SMAD2 antibody (51–1300, Thermo Fisher), anti-phospho-SMAD2 antibody (44–244G, Thermo Fisher), at 1:1000 in 5% BSA at 4 °C overnight on a rocking platform. After washing, the blots were incubated with sheep anti-mouse IgG-HRP antibody (NA9310V, GE Healthcare Life Sciences, England) and sheep anti-rabbit IgG-HRP antibody (NA9340V, GE Healthcare Life Sciences, England) at 1:2000 dilution. Then, chemiluminescent detection was performed using ECL Western Blotting Detection Regents (RPN2209, GE Healthcare Life Sciences). Signals were visualized and quantified by Fusion solo S system (Vilber Lourmat, France). In order to examine expression of additional proteins, antibodies were removed by incubating blots in stripping buffer (62.5 mM Tris, pH 6.7, 2% SDS, and 100 mM β-mercaptoethanol) for 30 min at 50 °C, blocked, and incubated with the next antibody.

### Cell transplantation in the mouse ischemic hindlimb model

The surgical treatment was based on previously established methods^16^. Briefly, an ischemic limb was created in the left leg of 7-week-old SCID mice under anaesthetic conditions using isoflurane. The external iliac artery and femoral artery were sutured with a 5–0 polypropylene thread. Six hours after surgery, saline (control group; 100 μl, n = 10), fibroblasts (fibroblast group; 10^5^ cells/100 μl saline/body, n = 10), DFAT cells (DFAT group; 10^5^ cells/100 μl saline/body, n = 10), and ASCs (ASC group; 10^5^ cells/100 μl saline/body, n = 10), were injected into the muscle of the femur of the ischemic limb. The amounts of injected cells (10^5^ cells/body) were determined as an optical dose by preliminary experiments. The ratio of blood flow between the healthy and ischemic limbs were measured using a blood perfusion imager (PeriScan, PERIMED, Sweden) at 7, 14, 21, and 28 days after injection. In addition, 28 days after injection, the ischemic limb was excised, fixed in 4% paraformaldehyde, and stained with IB4 and ASMA. The percentage of IB4- and ASMA-positive vessels were calculated in 5 different optical fields per ischemic limb.

### Statistical analysis

The assay results obtained in the experiments were expressed as mean ± SD. Two group comparisons were performed using Wilcoxon analysis. Multigroup comparisons were performed by one-way analysis of variance (ANOVA), followed by post-hoc statistical analysis of each group using the Tukey Kramer test. *P* < 0.05 was considered significant. Statistical analysis was performed using JMP, version 14 (SAS Institute Inc., Cary, NC).

## Results

### Characterization of DFAT cells in mice

**Figure 1:**
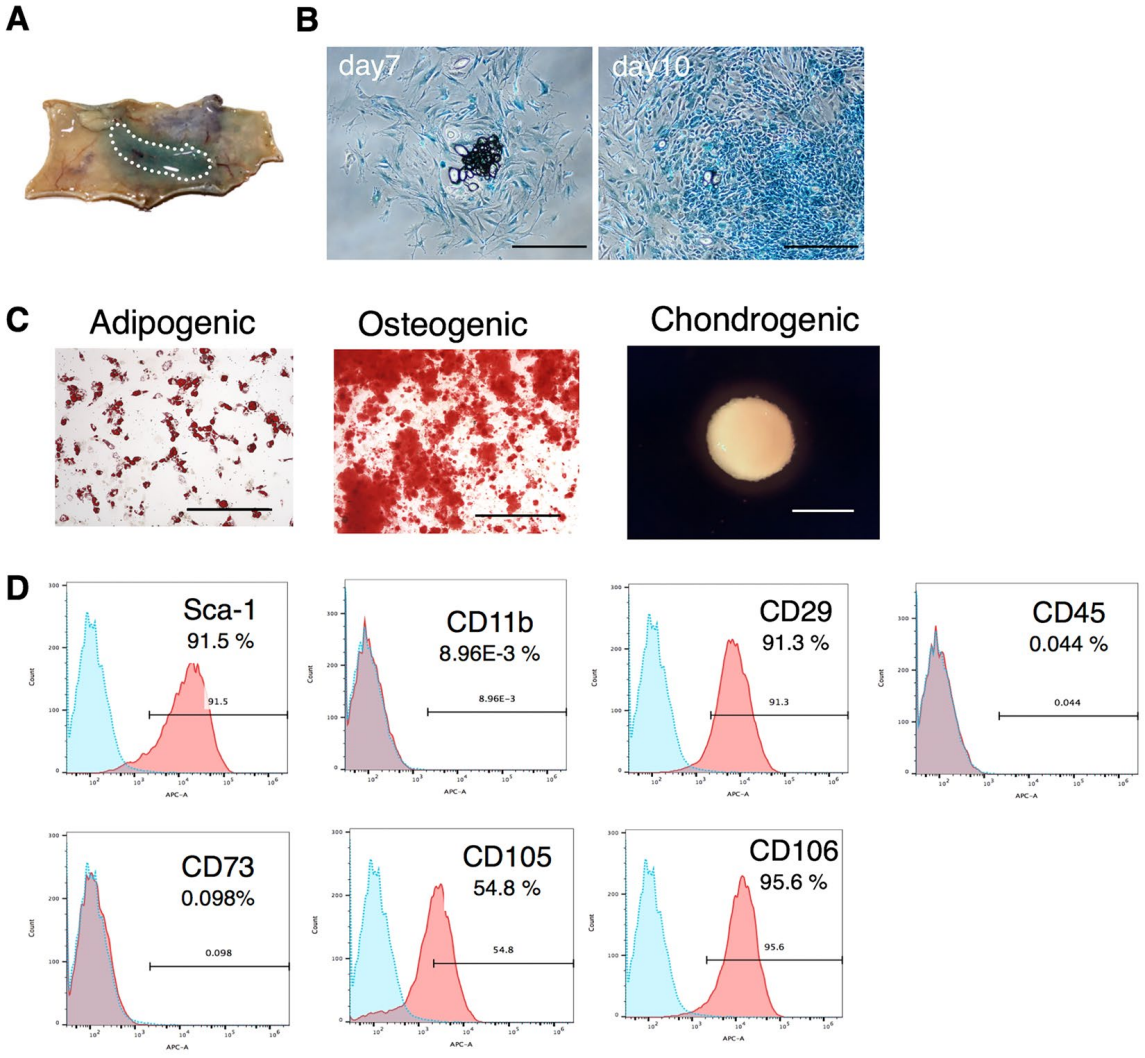
Characterization of mouse DFAT cells. (**A**) Subcutaneous adipose tissue of Fabp4-Cre/LacZ-ROSA (R26R) mice treated with 5-Bromo-4-chloro-1H-indol-3-yl β-D-galactopyranoside (X-gal). (**B**) DFAT cells from Fabp4-Cre/LacZ-ROSA (R26R) mice formed colonies with positive βgal activity on days 7 and 10. (**C**) Adipogenic differentiation (Oil red O, Scale = 200 μm), osteogenic differentiation (Alizarin red S, Scale = 200 μm), and chondrogenic differentiation (pellets, Scale = 500 μm) of DFAT cells. (**D**) Phenotypic analysis of DFAT cells using flow cytometry.

DFAT cells from Fabp4-Cre/LacZ-ROSA (R26R) mice were determined to be plastic-adherent and formed colonies which showed positive βgal activity. This indicated that DFAT cells were mostly derived from Fabp4 expressing adipose cells (Fig. 1A,B). In addition, DFAT cells showed adipogenic, osteogenic, and chondrogenic differentiation in each induction medium (Fig. 1C). Furthermore, phenotypic analysis of DFAT cells using flow cytometry revealed that the expression of Sca1, CD29, CD105, and CD106 were positive, whereas that of CD11b, CD45 and CD73 were negative (Fig. 1D).

### DFAT cells promote neovascularization in the hindlimb ischemia model

**Figure 2:**
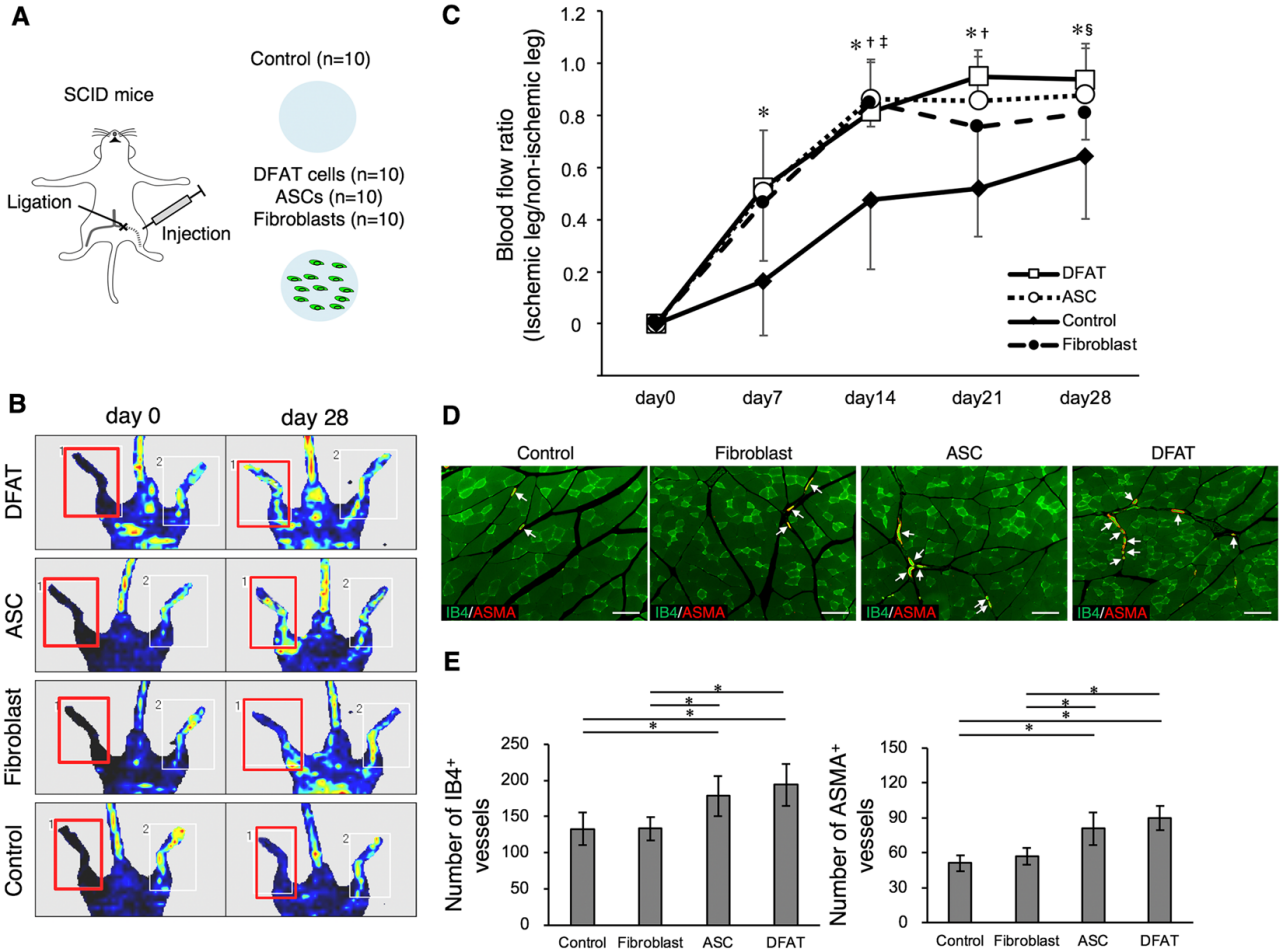
DFAT cells promote neovascularization in the ischemic hindlimb model. (**A**) A schematic illustration of the ischemic hindlimb model in SCID mice. The left femoral artery was ligated and injected with: saline (control group; n = 10), fibroblasts (fibroblast group; n = 10), ASCs (control group; n = 10) or DFAT cells (DFAT group; n = 10). (**B**) Representative images of blood flow in each group. The red boxes indicate the ischemic area evaluated. (**C**) The percentage of ischemic change in each group. *p < 0.05 DFAT cells vs. control, ^†^*p* < 0.05 ASCs vs. control, ^‡^*p* < 0.05 fibroblasts vs. control, ^§^*p* < 0.05 DFAT cells vs. fibroblasts. Values are represented as mean ± SD. (**D**) Representative photomicrographs of ischemic muscle tissue in each group. Arrows indicate IB4- and ASMA-double positive vessels. Scale = 100 μm. (**E**) The number of IB4 or ASMA-positive blood vessels in each group. Values are represented as mean ± SD. *p < 0.05.

In the hindlimb ischemia model of SCID mice, DFAT cells (1 ×10^5^ cells/body) were injected into ischemic muscle tissue and recovery of blood flow was evaluated and compared to other type of cells (Fig. 2A). Improvement of the blood flow ratio in the DFAT group was significantly higher than that of the control group from day 7 to day 28 after transplantation (Fig. 2B,C). Although no difference was recorded between the DFAT group and ASC group, only the DFAT group exhibited a significant difference from the control group during the 28 days. Finally, blood flow ratio increased by 0.94 in the DFAT group, 0.88 in the ASC group, 0.81 in the fibroblast group, and 0.64 in the control group at 28 days after transplantation. Immunohistological evaluation showed that the number of blood vessels stained by IB4 and ASMA were also significantly higher in the DFAT and ASC groups, compared with the control and fibroblast groups (Fig. 2D,E). These findings indicate that DFAT cells promote neovascularization and vessel maturation in the hindlimb ischemia model.

### DFAT cells secrete angiogenic factors

**Figure 3:**
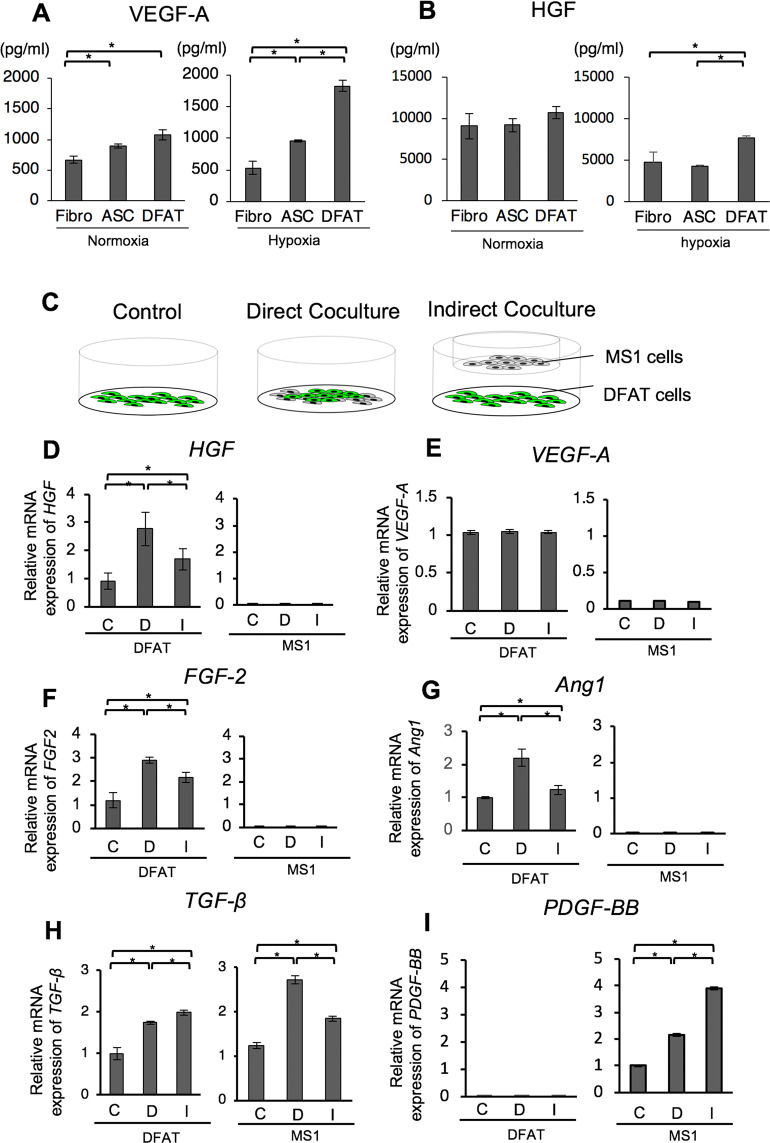
DFAT cells secrete several angiogenic factors. (**A**,**B**) The VEGF-A and HGF concentrations in the cell culture supernatant. The supernatants were collected from each of the cell cultures 48 hours after incubation under normal or hypoxic (1% O_2_) conditions. The levels of VEGF-A (**A**) and HGF (**B**) were then measured using ELISA. (**C**) Schematic illustrations of the co-culture experiments. (**D**–**I**) The gene expression of angiogenic factors in DFAT and MS1 cells. DFAT cells were co-cultured with MS1cells, alongside a control, for 72 hours. The mRNA levels of HGF (**D**), VEGF-A (**E**), FGF-2 (**F**), Ang1 (**G**), TGF-β1 (**H**), and PDGF-BB (**I**) were then measured by quantitative PCR. DFAT cells were cultured either alone (C: control group), co-cultured with MS1 cells directly (D: direct coculture group), or indirectly (I: indirect co-culture group). Values are represented as mean ± SD. *p < 0.05.

ELISA showed that the secretion of VEGF-A in DFAT cells and ASCs was significantly higher than that of fibroblasts under normoxic conditions (Fig. 3A). Under hypoxic conditions, the secretion of VEGF-A in DFAT cells was seen to increase significantly higher than that in ASCs and fibroblasts. The secretion of HGF in fibroblasts, ASCs, and DFAT cells were equal under normoxic conditions (Fig. 3B). However, under hypoxic conditions, the secretion of HGF in DFAT cells was much higher than that in ASCs and fibroblasts.

Next, we performed a co-culture experiment of vascular endothelial cells and DFAT cells (Fig. 3C). The gene expression of HGF, FGF-2, and Ang1 in DFAT cells was significantly higher in the indirect co-culture with vascular endothelial cells than that with the control (Fig. 3D,F,G). The expression of VEGF-A in DFAT cells did not change upon co-culturing with vascular endothelial cells (Fig. 3E). The expression of these angiogenic factors from the vascular endothelial cells was considerably lower than that of the DFAT cells (Fig. 3D–G). We also investigated other cytokines related to differentiation and recruitment of pericytes. The gene expression of TGFβ in DFAT cells and endothelial cells was significantly higher upon co-culturing (Fig. 3H). The expression of PDGF-BB in endothelial cells was significantly higher in the co-culture, but the expression in DFAT cells was barely detected (Fig. 3I). These data suggest that co-culture of DFAT cells with endothelial cells promote expression of several angiogenic factors in each type of cells.

### DFAT cells promote endothelial tube formation *in vitro*

**Figure 4:**
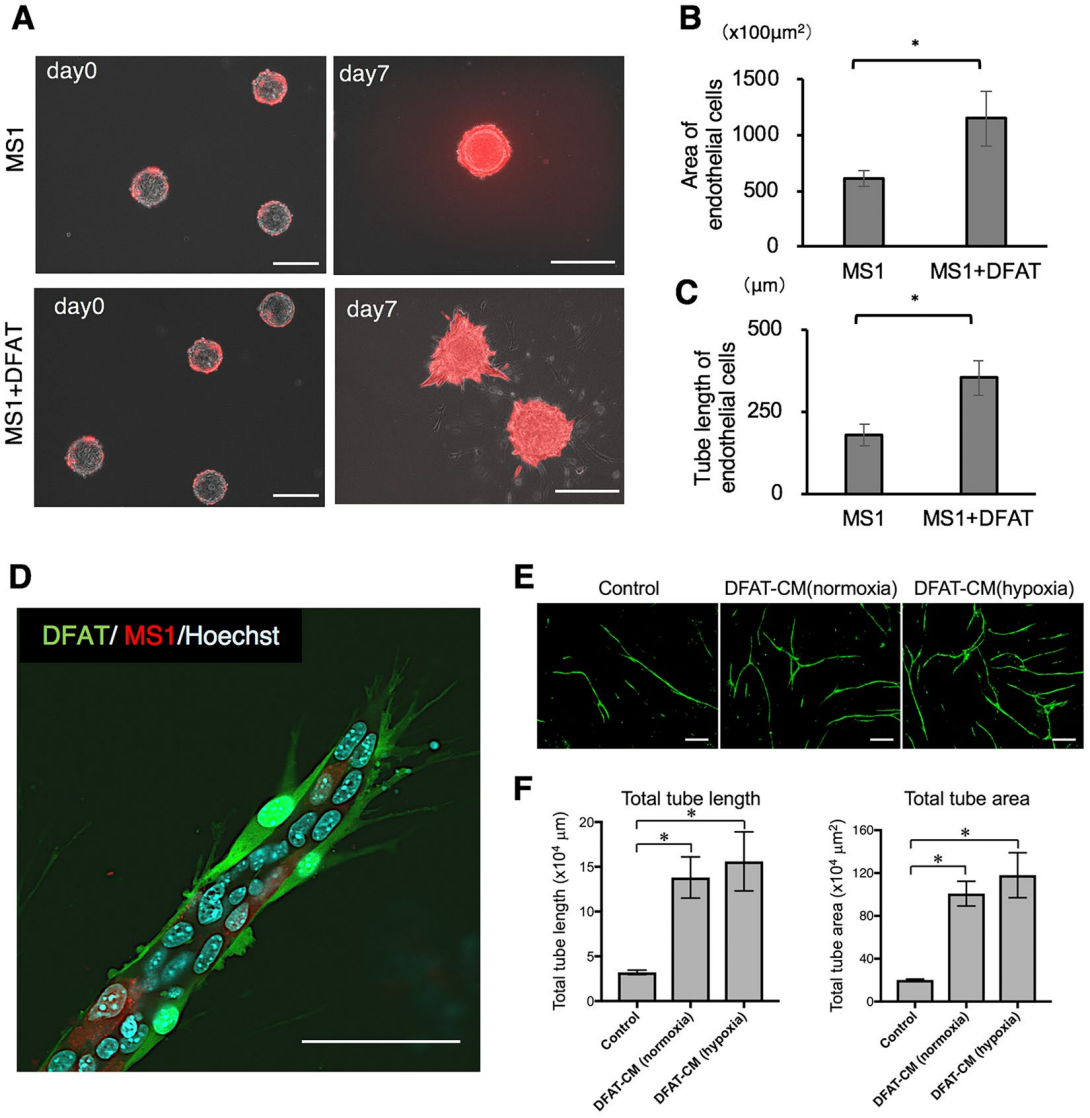
DFAT cells promote endothelial cell tube formation *in vitro*. (**A**) Representative images of collagen beads with mCherry-labelled MS1 cells in the collagen gel 3D culture with DFAT cells on days 0 and 7. Scale = 200 μm. (**B**,**C**) Quantification of the area (**B**) and the tube length (**C**) of DsRed-positive MS1 cells. (**D**) Fluorescence microscopic image of the tubules of DsRed-labelled MS1 cells with GFP-labelled DFAT cells. Scale = 200 μm. (**E**,**F**) The effect of DFAT cell conditioned media on tube formation ability in human umbilical vein endothelial cells were examined. Representative photomicrographs of tube-like structures in each group. Scale = 200 μm (**E**). Quantification of total tube length and total tube area in each group (**F**). Values are represented as mean ± SD. *p < 0.05.

In the collagen beads assay, DsRed-labelled MS1 cells exhibited tubular structure formation. Quantitative analysis revealed that the length and area of the tubules in the MS1 + DFAT group were significantly higher than that of the MS1 group on day 7 (Fig. 4A–C). GFP-labelled DFAT cells adhered to the outer surface of the tubules of the MS1 cells (Fig. 4D). Furthermore, DFAT cell conditioned media prepared from both normoxic and hypoxic culture conditions significantly promoted tube formation in human umbilical vein endothelial cells (HUVECs) (Fig. 4E,F). These results suggest that DFAT cells contribute endothelial cell tubular morphogenesis directly by differentiating vascular component cells as well as indirectly by the secretion of angiogenic factors.

### DFAT cells express pericyte markers upon co-culturing with endothelial cells

**Figure 5:**
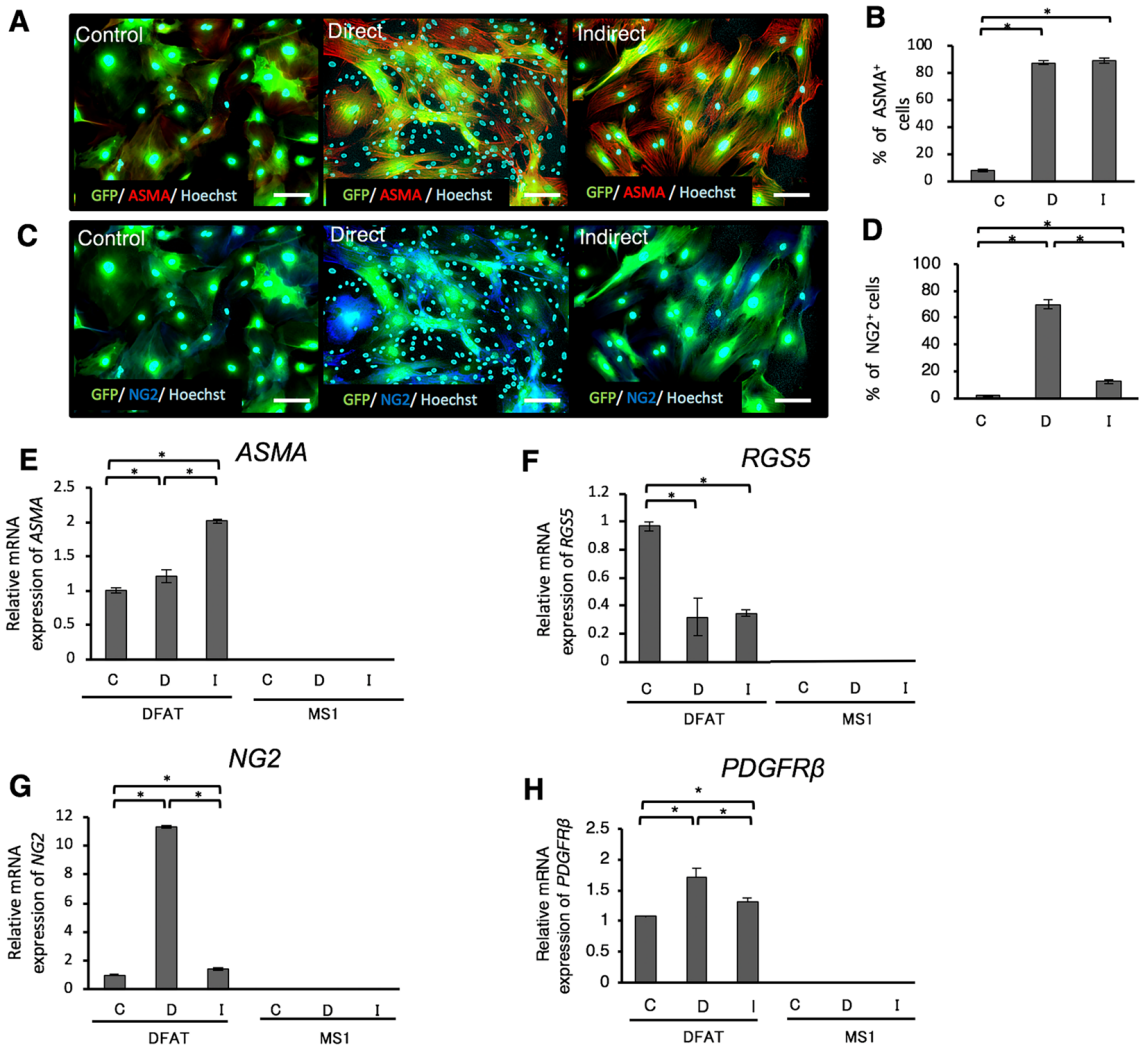
DFAT cells express pericyte markers in the co-culture with endothelial cells. (**A**,**C**) Immunofluorescence staining of GFP-labelled DFAT cells with ASMA (**A**) and NG2 (**C**) in each of the groups. Scale = 40 μm. (**B**,**D**) The percentage of ASMA-positive (**B**) and NG2-positive (**D**) cells. (**E**–**G**) The gene expression of ASMA (**E**), RGS5 (**F**), NG2 (**G**), and PDGFRβ (**H**) in DFAT and MS1 cells. DFAT cells were cultured alone (C: control group), co-cultured with MS1 cells directly (D: direct co-culture group), or indirectly (I: indirect co-culture group). Values are represented as mean ± SD. *p < 0.05.

The immunofluorescence staining results suggested that the levels of ASMA- and NG2-positive DFAT cells were significantly higher in the co-culture with MS1 cells, than that of the control (Fig. 5A,C). ASMA-positive DFAT cells increased significantly in both the direct and indirect co-culture groups (Fig. 5B). The number of NG2-positive DFAT cells in the direct co-culture was significantly higher than that in the indirect co-culture and control (Fig. 5D). The gene expression of mature pericyte-specific markers, ASMA, NG2, and PDGFRβ in DFAT cells was significantly higher upon co-culturing with endothelial cells compared with that of the control (Fig. 5E,G,H). Conversely, the levels of RGS5, an immature pericyte marker, significantly decreased upon co-culturing (Fig. 5F). The gene expression of such pericyte markers in endothelial cells is much lower than that in DFAT cells (Fig. 5E–H).

### Smad2/3 activation plays an important role in differentiation of DFAT cells into pericyte-like cells

**Figure 6:**
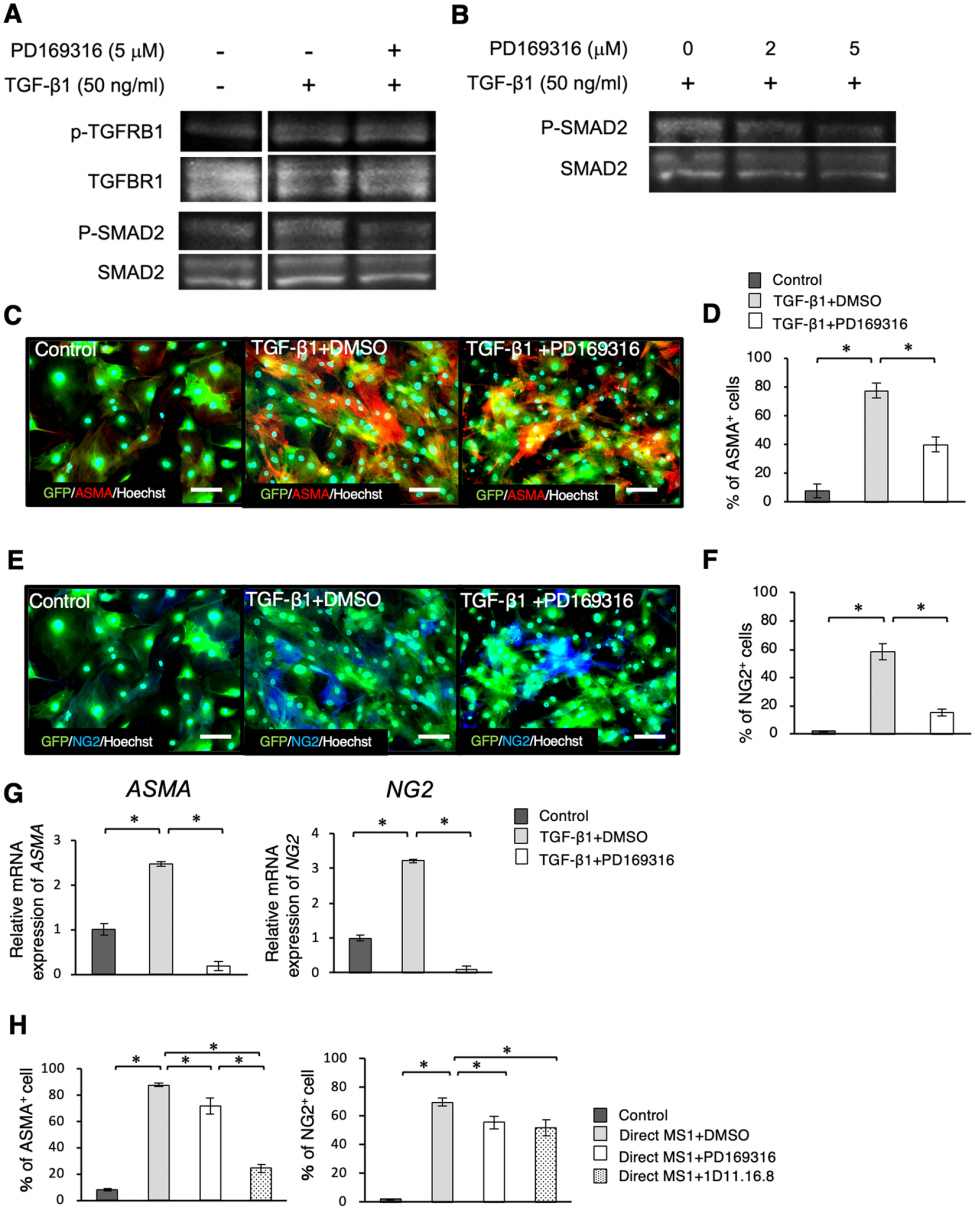
Smad2/3 activation plays an important role in differentiation of DFAT cells into mural cells. (**A**,**B**) Western blotting analysis for phospho-TGFBR1 and phospho-Smad2 expression in DFAT cells. Protein expression of phospho-TGFBR1, phospho-Smad2 in DFAT cells in the presence of TGF-β1 and a Smad2/3 inhibitor PD169316 (**A**). Effects of PD169316 in different doses on phospho-Smad2 in DFAT cells (**B**). (**C**–**G**) The changes of pericyte marker expressions in DFAT cells on adding DMSO or PD169316 into the culture medium was examined with TGF-β1. Immunofluorescence staining of GFP labelled DFAT cells with ASMA (**C**) and NG2 (**E**) in each of the groups. Scale = 30 μm. The percentage of ASMA-positive (**D**) and NG2-positive (**F**) cells. The gene expression of ASMA and NG2 in DFAT cells in the presence of TGF-β1 and PD169316 (**G**). (**H**) The changes of pericyte marker expressions in DFAT cells in the presence of a Smad2/3 inhibitor or a TGF-β1-neutralizing antibody, in co-culture with MS1 cells. The percentage of ASMA or NG2-positive cells (H). Values are represented as mean ± SD. *p < 0.05.

It was hypothesized that DFAT cells could be differentiating into pericytes through TGF-β1 signal transduction pathway. Then, we investigated the changes of pericyte marker expression in DFAT cells due to the addition of TGF-β1 and the Smad2/3 inhibitor. We confirmed that administration of 50 ng/ml TGF-β1 led phosphorylation of TGFBR1 and Smad2 in DFAT cells (Fig. 6A). The phosphorylation of Smad2 was inhibited in the presence of the Smad2/3 inhibitor PD169316 at the concentration of 2–5 μM (Fig. 6A,B). The expression of both ASMA and NG2, increased with the addition of TGF-β1 and was significantly suppressed by the Smad2/3 inhibitor as assessed by the immunofluorescence staining (Fig. 6C–F) and the gene expression analysis (Fig. 6G).

Additionally, the effect of pericyte markers on the induction of both, the Smad2/3 inhibitor and the TGF-β1-neutralizing antibody, were investigated in a co-culture with MS1 cells. The proportion of ASMA- and NG2-positive cells induced in the direct co-culture was significantly higher (Fig. 6H). The number of ASMA-positive cells was partially lower when treated with the Smad2/3 inhibitor, and decreased steadily on treatment with the TGF-β1-neutralizing antibody. On the other hand, the number of NG2-positive cells partially decreased upon treatment with both, the Smad2/3 inhibitor and the TGF-β1-neutralizing antibody. The results also suggested that the Smad2/3 pathway plays an important role in the differentiation of DFAT cells into pericyte-like cells.

### DFAT cells differentiate into pericyte-like cells in Matrigel

**Figure 7:**
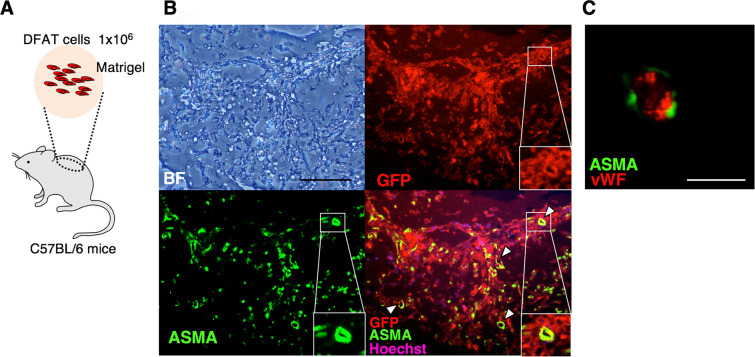
DFAT cells differentiated to mural cells in Matrigel. (**A**) Schematic illustration of Matrigel plague assay. GFP-labelled DFAT cells embedded in Matrigel were subcutaneously injected into the cervical area of 10-week-old male C57BL/6 mice. (**B**) Immunofluorescence staining of the Matrigel with GFP, ASMA, and Hoechst at 14 days after the gel injection. The arrowheads indicate ASMA- and GFP-double positive vessels. Scale = 200 μm. (**C**) Immunofluorescence staining of the Matrigel with ASMA and vWF 14 days after the gel injection. Scale = 100 μm.

We next performed a Matrigel plug assay to assess the differentiation property of DFAT cells in C57bL/6 mice (Fig. 7A). In transplanted Matrigel, GFP and ASMA-double positive cells were observed along the short axis of the vascular structure, suggesting DFAT cells differentiated into pericyte-like cells (Fig. 7B). In addition, vWF-positive endothelial cells were detected inside the lumen of the ASMA-positive DFAT cells (Fig. 7C). These findings suggest that DFAT cells differentiate into pericyte-like cells in the transplanted Matrigel.

## Discussion

Although it has been reported that DFAT cells are derived from mature adipocytes, to date, there are no reports which directly prove this. In this study, we performed genetic lineage tracing using LacZ mice and clarified that DFAT cells are derived from adipocytes expressing FABP4 (Fig. 1A,B). Furthermore, as a result of analysing the surface markers of mouse-derived DFAT cells using flow cytometry (Fig. 1D), MSC minimum criteria was almost met^17^. It has been reported that DFAT cells have a similar cell surface antigen expression profile as BM-MSCs and ASCs^12,18–20^, and the results of this study were aligned with this.

The mouse ischemic hindlimb model was then prepared and the therapeutic angiogenic effects of DFAT cells were compared with those of fibroblasts and ASCs (Fig. 2). The results confirmed that there was a significant improvement in the blood flow and an increase in the mature blood vessel density in the group which was administered DFAT cells, and only this group maintained a significant difference from the control group throughout the course of the experiment. To date, there are no reports that directly compare the angiogenic effects of DFAT cells with ASCs in a cell transplant in an ischemic limb. The improvement in the rate of ischemia and the degree of the blood vessel induction was about 1.5–2 times, which was aligned with the results of a previous report in the administration of stromal-vascular fraction cells from adipose tissue^21^.

The role of the paracrine signalling in the angiogenic effect of DFAT cells was then examined. The results indicated that the basal secretion of VEGF and HGF were higher in DFAT cells than that of ASCs in the hypoxic condition (Fig. 3A,B). Since we reported that DFAT cells are more homogeneous MSC-like cell population compared to ASCs^12^, it is speculated that purity of stem-like cell population is involved in the VEGF-A secretion ability and hypoxia-inducible factor (HIF) response. The cell transplantation site is normally considered to be within a hypoxic environment; this *in vitro* model shows that DFAT cells maintain a high level of secretory capacity even under conditions similar to that of a transplant site.

We also found that expression of several angiogenic factor genes including HGF, FGF-2, Ang1, and TGF-β from DFAT cells was enhanced by direct and indirect co-culture with vascular endothelial cells (Fig. 3D–G). Interestingly, co-culture with endothelial cells did not affect VEGF-A gene expression in DFAT cells, suggesting VEGF-A expression may be strictly regulated by specific stimuli such as hypoxia. Among the angiogenic factors expressed by DFAT cells, VEGF-A, FGF-2 and HGF were known to contribute mainly towards vascular endothelial cell proliferation and differentiation (tubular morphogenesis). Our experimental data indicate that endothelial cell tube formation was promoted not only by direct co-culture with DFAT cells but also by administration of DFAT cell conditioned medium (Fig. 4E,F). These findings suggest the secreted angiogenic factors plays an important role in neovascularization of endothelial cells.

It is well known that PDGF-BB play a role in pericyte recruitment and Ang1 and TGF-β mainly contributed towards pericyte maturation. From the above results, an estimated mechanism of blood flow improvement by DFAT cell transplantation in the ischemic hindlimb model is as follows; VEGF-A and HGF, secreted by transplanted DFAT cells in the hypoxic environment, act on vascular endothelial cells to promote angiogenesis. Then PDGF-BB, secreted from the vascular endothelial cells, induces the recruitment of the surrounding pericytes. Finally, Ang1 secreted by DFAT cells and TGF-β secreted by both DFAT cells and vascular endothelial cells, allow the pericyte to mature and the vessels to become functional.

Furthermore, the possibility that DFAT cells differentiate into vascular component cells, specifically pericytes, was examined. First, in the three-dimensional co-culture of DFAT cells and vascular endothelial cells, DFAT cells existed and covered the outer structure of the vascular endothelial tube (Fig. 4D). In the co-culture with vascular endothelial cells, the expression of NG2 and ASMA, reported as pericyte markers, increased at the protein and mRNA levels (Fig. 5A–H). Moreover, when TGF-β1 was added to DFAT cells, the expression of NG2 and ASMA increased. Conversely, with the addition of the Smad2/3 inhibitor, their expression was noted to decrease (Fig. 6C–G). Lower gene expressions of ASMA and NG2, compared with the base line, were observed (Fig. 6G), and this was thought to be as a result of autocrine inhibition. On the other hand, the pericyte marker expression in the direct co-culture with vascular endothelial cells was only partially suppressed by the Smad2/3 inhibitor and the TGF-β1 neutralizing antibody (Fig. 6H). This may be affected by direct cell-cell interactions such as Notch signalling rather than TGFβ1 signalling, and is a subject which has potential to be studied in the future.

In this study, DFAT cells were seen to acquire pericyte-like traits upon co-culturing with vascular endothelial cells with the addition of TGF-β1. On the other hand, previous studies have reported that a myofibroblast-like traits were acquired by the DFAT cells upon interaction with TGF-β1^22^. Myofibroblasts mainly originate from fibroblasts and mesenchymal stem cells and play a major role in the pathogenesis of fibrosis induced after inflammation, such as wound healing^23^. The plasticity of pericytes and myofibroblasts under fibrotic conditions has also been reported^24,25^, but their distinction was difficult.

To trace the path of DFAT cells at the site of transplantation, GFP-labelled DFAT cells were transplanted to the mouse ischemic hindlimb model (data not shown). However, the identification of DFAT cells that acquired pericyte-like traits could not be ascertained. There remains a possibility that the pericytes were replaced by a host-derived cell, and so this observation was examined in the ischemic hindlimb model. Alternatively, it could be stated that GFP-labelled DFAT cells existed around the blood vessels as ASMA-positive cells in the Matrigel plug assay (Fig. 7).

In this series of experiments, we assessed the potential therapeutic angiogenetic effect of DFAT cells via the secretion of angiogenic factors and the promotion of pericyte differentiation via the TGF-β1-Smad2/3 signal transduction pathway. DFAT cells can be prepared very efficiently and with minimal invasiveness, and can be expected to be used as a source of transplanted cells in future cell therapies. At the same time, it is hoped that the detailed mechanism of the angiogenesis of DFAT cells will be elucidated by further studies.

## Conclusion

In this study, we showed that the angiogenesis and maturation effects of DFAT cells was more effective than that of other cells. Furthermore, the possibility of DFAT cells differentiating into pericyte-like cells upon interaction with vascular endothelial cells was proven. Using a differentiation mechanism, it was also revealed that the Smad2/3 signalling pathway, via TGF-β1 stimulation, is key to the process. DFAT cells can be prepared with minimal invasiveness and high efficiency, and are expected to become a source for transplanted cells in future cell-based therapy.

## Supplementary information

Supplementary information.
