# Supplementary material for: The neovascularization effect of dedifferentiated fat cells
Source: Sci Rep. 2020 Jun 8;10:9211. doi: 10.1038/s41598-020-66135-1 (PMC7280264; doi:10.1038/s41598-020-66135-1)
Supplement: Supplementary file 1 — Supplementary information. [file 41598_2020_66135_MOESM1_ESM.docx]

**Title Page**

**Title**: **The neovascularization effect of dedifferentiated fat cells**

Hirofumi Watanabe ^a,1^: [watanabe.hirofumi@nihon-u.ac.jp](mailto:watanabe.hirofumi@nihon-u.ac.jp)

Shunpei Goto^b,1^: [goto.shumpei@nihon-u.ac.jp](mailto:goto.shumpei@nihon-u.ac.jp)

Reona Kato^b^: takeiteasy232323@gmail.com

Shogo Komiyama^c^: kommy.sho@gmail.com

Yuki Nagaoka^c^: nagaoka.yuki@nihon-u.ac.jp

Tomohiko Kazama^c^: kazama.tomohiko@nihon-u.ac.jp

Chii Yamamoto^c^: yamamoto.chii@nihon-u.ac.jp

Li Yuxin^c^: li.yuxin@nihon-u.ac.jp

Noriyoshi Konuma ^b^: konuma.noriyoshi@nihon-u.ac.jp

Kazuhiro Hagikura^c^: [kazuhiro.hagikura@gmail.com](mailto:kazuhiro.hagikura@gmail.com)

Taro Matsumoto^c^**^*^**: matsumoto.taro@nihon-u.ac.jp

^1^H. Watanabe and S. Goto contributed equally to this manuscript and are shared first authors.

^a^Department of Pediatrics and Child Health, Nihon University School of Medicine, Tokyo, Japan

^b^Department of Pediatric Surgery, Nihon University School of Medicine, Tokyo, Japan

^c^Department of Functional Morphology, Division of Cell Regeneration and Transplantation, Nihon University School of Medicine, Tokyo, Japan

***Corresponding author:** Taro Matsumoto

Department of Functional Morphology, Division of Cell Regeneration and Transplantation,  Nihon University School of Medicine, Tokyo, Japan

30-1 Kami-cho Ooyaguchi, Itabashi-ku, Tokyo 173-8610, Japan

Tel: +81-3-3972-8111 (ext. 2703); Fax: +81-3-3972-8666

E-mail: matsumoto.taro@nihon-u.ac.jp

**Supplementary Information**

**The neovascularization effect of dedifferentiated fat cells**

Hirofumi Watanabe ^a^, Shunpei Goto^b^, Reona Kato^b^, Shogo Komiyama^c^, Yuki Nagaoka^c^, Tomohiko Kazama^c^, Chii Yamamoto^c^, Li Yuxin^c^, Noriyoshi Konuma ^b^, Kazuhiro Hagikura^c^, Taro Matsumoto^c^**^*^**

^a^Department of Pediatrics and Child Health, Nihon University School of Medicine, Tokyo, Japan

^b^Department of Pediatric Surgery, Nihon University School of Medicine, Tokyo, Japan

^c^Department of Functional Morphology, Division of Cell Regeneration and Transplantation, Nihon University School of Medicine, Tokyo, Japan

*Corresponding author

E-mail: matsumoto.taro@nihon-u.ac.jp

Supplementary Figure 1:


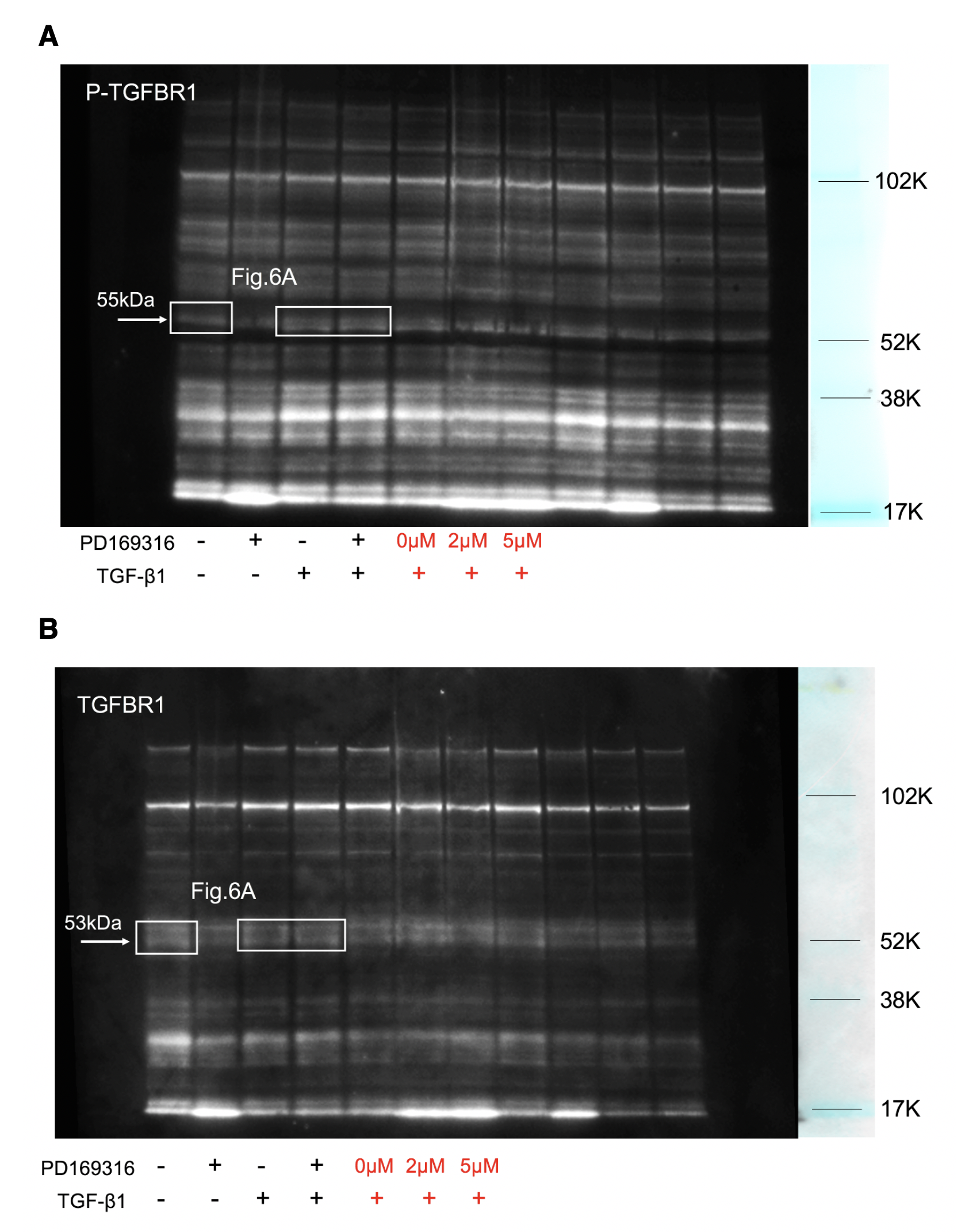


**Supplementary Fig. 1:** Full-length western blots for cropped images in Fig. 6A. The blots were probed with antibodies for phospho-TGFBR1 (A) and TGFBR1 (B). Arrows indicate the bands of interest.

Supplementary Figure 2:
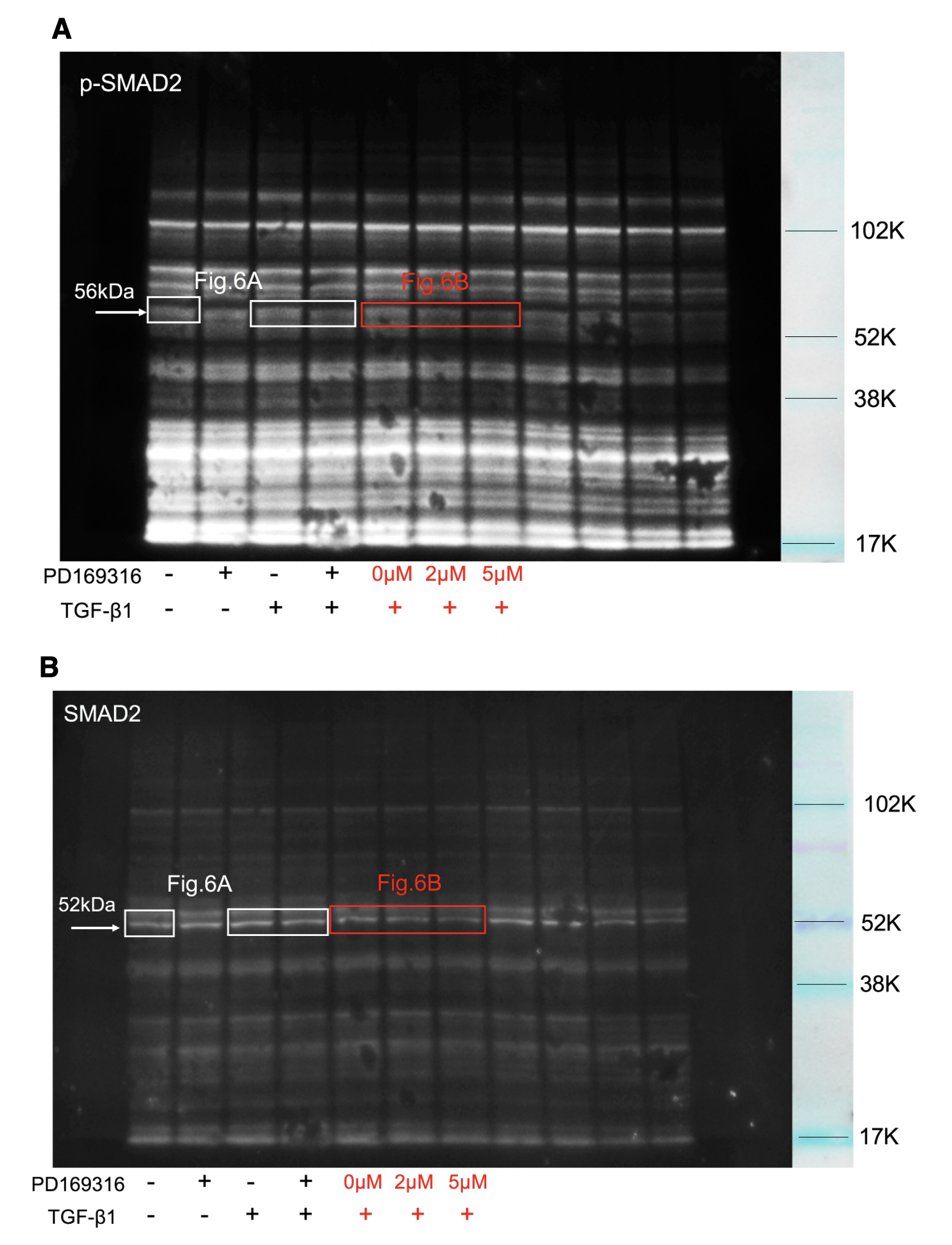
 **Supplementary Fig. 2:** Full-length western blots for cropped images in Fig. 6A and 6B. The blots were probed with antibodies for phospho-SMAD2 (A) and SMAD2 (B). Arrows indicate the bands of interest.
